# Myocardial protective effects of a c‐Jun N‐terminal kinase inhibitor in rats with brain death

**DOI:** 10.1111/jcmm.12676

**Published:** 2016-04-12

**Authors:** Wenzhi Guo, Shengli Cao, Bing Yan, Gong Zhang, Jie Li, Yongfu Zhao, Shuijun Zhang

**Affiliations:** ^1^ Department of Hepatobiliary and Pancreatic SurgeryThe First Affiliated Hospital of Zhengzhou UniversityZhengzhouHenanChina; ^2^ Henan Key Laboratory of Digestive Organ TransplantationZhengzhouHenanChina

**Keywords:** heart transplantation, brain death, apoptosis, JNK inhibitor

## Abstract

To investigate whether the mitochondrial apoptotic pathway mediates myocardial cell injuries in rats under brain death (BD), and observe the effects and mechanisms of the c‐Jun N‐terminal kinase (JNK) inhibitor SP600125 on cell death in the heart. Forty healthy male Sprague‐Dawley (SD) rats were randomized into four groups: sham group (dural external catheter with no BD); BD group (maintain the induced BD state for 6 hrs); BD + SP600125 group (intraperitoneal injection of SP600125 10 mg/kg 1 hr before inducing BD, and maintain BD for 6 hrs); and BD + Dimethyl Sulphoxide (DMSO) group (intraperitoneal injection of DMSO 1 hr before inducing BD, and maintain BD for 6 hrs). Real‐time quantitative PCR was used to evaluate mRNA levels of Cyt‐c and caspase‐3. Western blot analysis was performed to examine the levels of mitochondrial apoptosis‐related proteins p‐JNK, Bcl‐2, Bax, Cyt‐c and Caspase‐3. TUNEL assay was employed to evaluate myocardial apoptosis. Compared with the sham group, the BD group exhibited increased mitochondrial apoptosis‐related gene expression, accompanied by the elevation of p‐JNK expression and myocardial apoptosis. As the vehicle control, DMSO had no treatment effects. The BD + SP600125 group had decreased p‐JNK expression, and reduced mitochondrial apoptosis‐related gene expression. Furthermore, the apoptosis rate of myocardial cells was reduced. The JNK inhibitor SP600125 could protect myocardial cells under BD through the inhibition of mitochondrial apoptosis‐related pathways.

Heart transplantation is an effective method to treat end‐stage heart diseases 1, 2, 3. The main factor restricting heart transplantation is the lack of suitable donors 4. Brain death (BD) donors have become a major source of organs for heart transplantation. However, roughly more than 25% of potential donors are discarded because of hemodynamic instability and loss of primary cardiac function. Brain death is a pathophysiological process. Clinical and experimental studies show that apoptosis of myocardial cells accounts for the disqualification of donor hearts 5, 6, 7, 8.

SP600125 is a commonly used and highly selective inhibitor for c‐Jun N‐terminal kinase (JNK). Prior studies have shown that SP600125 can reduce myocardial injury under ischaemia‐reperfusion 9, 10. However, it still elusive whether SP600125 can alleviate myocardial cell damage under the condition of BD.

## Materials and methods

### Animals and grouping

Healthy male SD rats weighing 200–250 g were supplied by the Experimental Animal Center of Henan Province. Forty SD rats were randomized into four groups: sham group (dural external catheter and no induction of BD); BD group (maintain induce BD for 6 hrs); BD + SP600125 group (intraperitoneal injection of SP600125 (10 mg/kg) 1 hr before inducing BD, and maintain BD for 6 hrs) 11.; and BD + DMSO group (intraperitoneal injection of DMSO 1 hr before inducing BD, and maintain BD for 6 hrs).

### Brain death model

The rat BD model was established by increasing intracranial pressure in a slow and intermittent way 12. Anaesthesia was performed by intraperitoneal injection of 1% sodium pentobarbital (0.6 ml/100 g). After anaesthesia was induced, tracheotomy was performed for mechanical ventilation after BD. The catheter was connected to the saphenous artery and tail vein to monitor the arterial blood pressure and establish the venous transfusion access. Cystostomy was performed to measure the amount of urine. On the front left of the skull's coronal and sagittal lines, a hole was drilled with a diameter of 4 mm and a Fogarty arterial embolectomy catheter was placed in the epidural area for saline injection. The pressure was increased by injection at a rate of 4 μl/min. until the occurrence of BD at approximately 240 μl. Brain death was confirmed by the criteria of: (*i*) absence of spontaneous respiration; (*ii*) flat EEG; and (*iii*) no brain stem reflex.

### Western blot

Rat heart tissue (100 mg) was lysed with 1 ml of Radio‐Immunoprecipitation Assay (RIPA) and 10 μl of Phenylmethanesulfonyl Fluoride (PMSF) in an eppendorf tube using an ultrasonic tissue disrupter. Protein concentrations were measured using the Bicinchoninic Acid (BCA) assay. Protein samples (40 μg) were separated by SDS‐PAGE gel electrophoresis and transferred onto a nitrocellulose membrane. Membranes were then blocked in 5% skim milk for 1 hr, followed by incubation with primary antibody against Cyt‐c (1:1000; Cell Signaling Technology, Inc., Shanghai, China), caspase‐3 (Cell Signaling Technology, Inc.), Bax, Bcl‐2 and p‐JNK overnight at 4°C. Membranes were washed three times with Tris Buffered Saline with Tween20 (TBST), followed by incubation with secondary antibody (1:5000; Zhongshan Golden Bridge, goat anti‐rabbit lgG‐HRP) for 1 hr. β‐actin (1:1000; Zhongshan Golden Bridge, Beijing, China) was used as the internal control. Specific bands were detected using an enhanced chemiluminescence system and captured on X‐ray film. Western blots were performed in at least three independent experiments. The density of the bands on the membrane was scanned and analysed with Quantity one software (Life Science Research, Education, Process Separations, Food Science, Hercules, California).

### Real‐time PCR

RNA was extracted from the cardiac muscle of rats using Trizol according to the manufacturer's instructions and measured by absorbance at A_260_/A_280_. cDNA was then synthesized by reverse transcription. Primer sequences for real‐time PCR are as follows: caspase‐3 forward 5′‐TTGCGCCATGCTGAAACTGTACG‐3′, reverse 5′‐AAAGTGGCGTCCAGGGAGAAGG‐3′; Cyt‐C forward 5′‐GGAGGCAAGCATAAGACTGG‐3′, reverse 5′‐GTCTGCCCTTTCTCCCTTCT‐3′; and internal control β‐actin forward 5′‐CTCTATCCTGGCCTCACTGTCCACC‐3′, and reverse 5′‐CTCTATCCTGGCCTCACTGTCCACC‐3′. The 25 μl total reaction mixture included 12.5 μl SYBR Green mix, 1 μl forward and reverse primers, 8 μl ddH_2_O, and 2.5 μl cDNA. The reaction conditions were 94°C for 30 sec. for one cycle; and 94°C for 10 sec., 55°C for 30 sec., and 72°C for 1 min., for 30 cycles. The 2^−ΔΔCT^ method was used to calculate the relative expression of mRNA.

### TUNEL assays

Myocardial tissue samples were formalin‐fixed, conventionally dehydrated and embedded. Serial sections (4 μm) were dewaxed and ethanol‐rehydrated. Samples were stained according to the TUNEL apoptosis kit instructions (In Situ Cell Death Detection Kit, Fluorescein; Roche, Indianapolis, USA).

### Statistical analysis

SPSS17.0 was used for statistical analysis, and all data were expressed as mean ± S.D. ($\overset{¯}{x}$ ± S). Comparisons among more than two groups were performed with one‐way anova followed by *post‐hoc* Bonferroni test, and the test levels α = 0.05 and *P* < 0.05 were considered statistically significant.

## Result

### mRNA expression levels of mitochondrial apoptosis‐related genes

**Figure 1:**
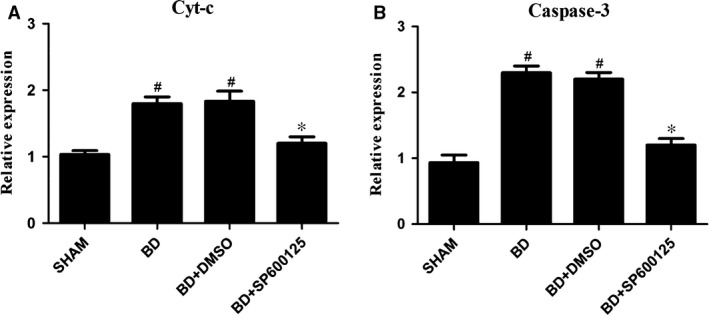
Effects of pretreatment with SP600125 on the myocardial mRNA expressions of Cyt‐c and caspase‐3 after 6 hrs of brain death. The mRNA expressions of Cyt‐c (**A**) and caspase‐3 (**B**) were analysed using quantitative PCR. All values shown are mean ± S.D. ^#^indicates *P* < 0.05 when compared to the sham group. *indicates *P* < 0.05 when compared to the BD group.

To examine whether SP600125 can alleviate myocardial cell damage under BD, we examined key apoptotic factors in a rat BD model. Real‐time PCR results showed that compared with the sham group, the BD group exhibited increased mRNA expression of Cyt‐c and caspase‐3 (*P* < 0.05). The BD + DMSO control group showed no difference in mRNA expression of Cyt‐c and caspase‐3 when compared to the BD group (*P* > 0.05). Notably, the BD + SP600125 group showed significant reduction in mRNA expression of Cyt‐c and caspase‐3 when compared to the BD group (*P* < 0.05; Fig. 1). These results suggest that the JNK inhibitor SP600125 down‐regulated the expression of mitochondrial apoptosis‐related genes such as Cyt‐c and caspase‐3.

### Expression levels of mitochondria‐related apoptotic proteins

**Figure 2:**
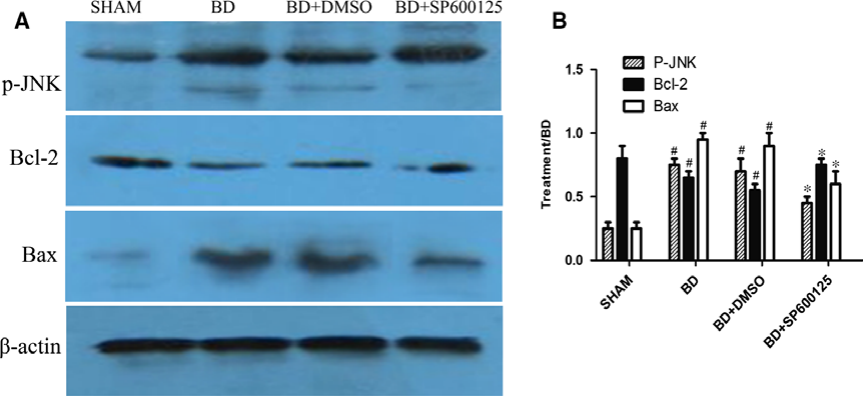
Effects of pretreatment with SP600125 on the myocardial protein expressions of p‐JNK, Bcl‐2 and Bax after 6 hrs of brain death. The protein expressions of p‐JNK, Bcl‐2 and Bax were analysed using Western blot (**A**) and normalized to β‐actin expression (**B**). All values shown are mean ± S.D. ^#^indicates *P* < 0.05 when compared to the sham group. *indicates *P* < 0.05 when compared to the BD group.

**Figure 3:**
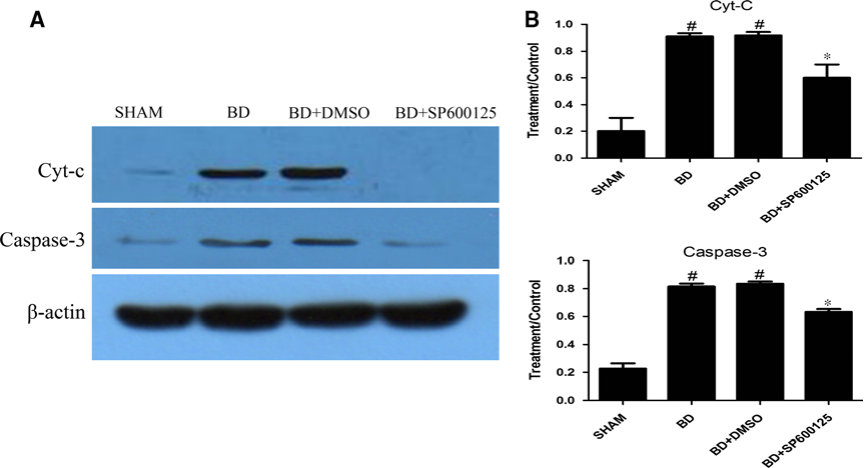
Effects of pretreatment with SP600125 on the myocardial protein expressions of Cyt‐c and caspase‐3 under brain death. The protein expressions of Cyt‐c and caspase‐3 were analysed using Western blot (**A**) and normalized to β‐actin expression (**B**). All values shown are mean ± S.D. ^#^indicates *P* < 0.05 when compared to the sham group. *indicates *P* < 0.05 when compared to the BD group.

To confirm the PCR results, we performed Western blot analysis to measure the protein levels in the myocardium. Compared to the sham group, the BD group exhibited increased expression in p‐JNK, Bax, Cyt‐c and caspase‐3, while Bcl‐2 expression was reduced (*P* < 0.05). Administration of DMSO (the BD + DMSO group) had no effects on mitochondria‐related apoptotic protein expression when compared to the BD group (*P* > 0.05). SP600125 significantly reduced the protein levels of p‐JNK, Bax, Cyt‐c and caspase‐3, and increased Bcl‐2 expression (*P* < 0.05; Figs 2 and 3).

### Evaluation of myocardial apoptosis

**Figure 4:**
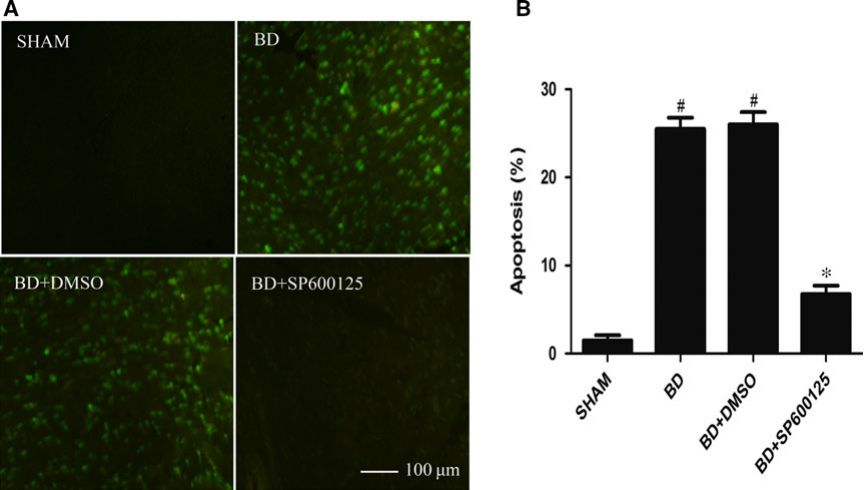
SP600125 reduces brain death‐induced apoptosis in heart. (**A**) Representative fluorescent micrographs showing positive TUNEL staining (green). (**B**) Pooled data showing the percentage of TUNEL‐positive cells in each group. All values shown are mean ± S.D. ^#^indicates *P* < 0.05 when compared to the sham group. *indicates *P* < 0.05 when compared to the BD group.

TUNEL assay showed that compared with the sham group, the BD group exhibited an increased apoptosis percentage of myocardial cells (*P* < 0.05). The BD + DMSO group showed no statistically significant effects on myocardial apoptosis when compared to the BD group (*P* > 0.05). Administration of SP600125 (the BD + SP600125 group) significantly reduced myocardial apoptosis (*P* < 0.05; Fig. 4).

## Discussion

Apoptosis is programmed cell death regulated by a series of caspases, a family of cysteine proteases 13. As an important member of the MAPK family, JNK has a wide range of biological activities, including several pro‐apoptotic functions. First, JNK up‐regulates the expression of pro‐apoptotic proteins. Activated JNK enhances the activity of transcription factor complex AP‐1, thus promoting the expression of p53, Bax, FasL, tumour necrosis factor and other pro‐apoptotic proteins. Second, JNK functions in the mitochondrial pathway 14, 15, 16 by prompting the release of Cyt‐C. Combined with caspase‐9/Apaf‐1, this process further leads to the activation of caspase‐3 which targets apoptotic substrates. Caspase‐3 is considered as a key executor of apoptosis by degrading several intracellular substrates (*e.g*. cytoskeletal proteins and nucleoprotein) 17. As a result, myocardial apoptosis accounts for the reduction of myocardial cells in myocarditis and dilated cardiomyopathy 18, 19, 20.

Heart transplantation is an effective treatment for end‐stage heart diseases. In recent years, the efficacy of heart transplantation has increased, but early complications, especially the functional failure of early primary graft, are still very prominent. After BD, the dramatic change in body hemodynamics results in myocardial ischaemia and hypoxia, and the sympathetic nervous system is activated to release large amounts of catecholamines. Metabolic disorders caused by endocrine imbalance and activation of large amounts of inflammatory mediators under stringent state induce myocardial apoptosis in BD. In this study, we found that after BD, Bax, Cyt‐c and caspase‐3 protein expression levels increased and Bcl‐2 expression decreased. Quantitative PCR confirmed that the mRNA expression of Cyt‐c and caspase‐3 also increased under BD. In addition, the TUNEL assay revealed increased myocardial apoptosis after BD. Together, these results indicate that the JNK signalling pathway mediates myocardial injury under BD *via* the mitochondrial apoptotic pathways.

SP600125 (also known as anthrapyrazolone or 1,9‐Pyrazoloanthrone) is a commonly used and highly selective inhibitor for JNK. In their study of myocardial ischaemic injury, Ferrandi *et al*. 21 found that JNK inhibition can reduce myocardial apoptosis in myocardial infarction. In this study, we have shown that administration of SP600125 resulted in significant reduction of p‐JNK expression, as well as the expression of Bax, Cyt‐c and caspase‐3. In contrast, Bcl‐2 expression was increased. In addition, quantitative PCR showed that the mRNA levels of Cyt‐C and caspase‐3 were significantly reduced by SP600125 treatment. Furthermore, the apoptosis of myocardial cells in BD rats was significantly reduced. Together, these results indicate that SP600125 can reduce apoptosis of myocardial cells by inhibiting JNK activity, and thus protect the heart under the state of BD.

The results of this study demonstrate the activation of the JNK signalling pathway mediates apoptosis of myocardial cells through the mitochondrial pathways in rats with BD. JNK inhibitor SP600125 can significantly reduce the myocardial apoptosis in BD rats. Out results provide the impetus to target the JNK signalling pathway for development of new drugs for heart protection. We anticipate our findings can help improve the quality of donor hearts as well as the prognosis of heart transplantation recipients.

## Conflicts of interest

The authors confirm that there are no conflicts of interest.

## References

[1] Smith TB , Shridhar P , Khalil R , *et al* Prolonged duration of transbrachial intra‐aortic balloon pump as bridge to heart transplantation. BMJ Case Rep. 2015; 2015: doi: 10.1136/bcr‐2014‐208658. 10.1136/bcr-2014-208658 PMC4330429 25657198

[2] Schweiger M , Stiasny B , Dave H , *et al* Pediatric heart transplantation. J Thorac Dis. 2015; 7: 552–9. 25922739 10.3978/j.issn.2072-1439.2015.01.38 PMC4387410

[3] Wilhelm MJ . Long‐term outcome following heart transplantation: current perspective. J Thorac Dis. 2015; 7: 549–51. 25922738 10.3978/j.issn.2072-1439.2015.01.46 PMC4387387

[4] Kuwaki K , Tseng YL , Dor FJ , *et al* Heart transplantation in baboons using alpha1, 3‐galactosyltransferase gene‐knockout pigs as donors: initial experience. Nat Med. 2005; 11: 29–31. 15619628 10.1038/nm1171

[5] Wilhelm MJ , Pratschke J , Beato F , *et al* Activation of the heart by donor brain death accelerates acute rejection after transplantation. Circulation. 2000; 102: 2426–33. 11067799 10.1161/01.cir.102.19.2426

[6] Novitzky D , Cooper DK , Reichart B . Hemodynamic and metabolic responses to hormonal therapy in brain‐dead potential organ donors. Transplantation. 1987; 43: 852–4. 3296351

[7] Kirsch M , Bertrand S , Lecerf L , *et al* Brain death‐induced myocardial dysfunction: a role for apoptosis? Transplant Proc. 1999; 31: 1713–4. 10331047 10.1016/s0041-1345(99)00073-1

[8] Szabo G , Hackert T , Sebening C , *et al* Role of neural and humoral factors in hyperdynamic reaction and cardiac dysfunction following brain death. J Heart Lung Transplant. 2000; 19: 683–93. 10930818 10.1016/s1053-2498(00)00129-7

[9] Qi D , Hu X , Wu X , *et al* Cardiac macrophage migration inhibitory factor inhibits JNK pathway activation and injury during ischemia/reperfusion. J Clin Invest. 2009; 119: 3807–16. 19920350 10.1172/JCI39738 PMC2786800

[10] Fiorillo C , Becatti M , Pensalfini A , *et al* Curcumin protects cardiac cells against ischemia‐reperfusion injury: effects on oxidative stress, NF‐kappaB, and JNK pathways. Free Radic Biol Med. 2008; 45: 839–46. 18638545 10.1016/j.freeradbiomed.2008.06.013

[11] Cao S , Wang T , Yan B , *et al* Protective effects of SP600125 inbrain death‐induced liver injury. Clin ResHepatol Gastroenterol. 2014; 38: 577–82. 10.1016/j.clinre.2014.05.004 24969683

[12] Zhang S , Cao S , Wang T , *et al* Modified brain death model for rats. Exp Clin Transplant. 2014; 12: 469–73. 24918972 10.6002/ect.2013.0229

[13] Lalaoui N , Lindqvist LM , Sandow JJ , *et al* The molecular relationships between apoptosis, autophagy and necroptosis. Semin Cell Dev Biol. 2015; 39: 63–9. 25736836 10.1016/j.semcdb.2015.02.003

[14] Liu J , Lin A . Role of JNK activation in apoptosis: a double‐edged sword. Cell Res. 2005; 15: 36–42. 15686625 10.1038/sj.cr.7290262

[15] Yu C , Minemoto Y , Zhang J , *et al* JNK suppresses apoptosis *via* phosphorylation of the proapoptotic Bcl‐2 family protein BAD. Mol Cell. 2004; 13: 329–40. 14967141 10.1016/s1097-2765(04)00028-0

[16] Chen F . JNK‐induced apoptosis, compensatory growth, and cancer stem cells. Cancer Res. 2012; 72: 379–86. 22253282 10.1158/0008-5472.CAN-11-1982 PMC3261582

[17] Singhal P , Kapasi A , Reddy K , *et al* Opiates promote T cell apoptosis through JNK and caspase pathway. Adv Exp Med Biol. 2001; 493: 127–35. 11727758 10.1007/0-306-47611-8_15

[18] Gottlieb RA , Burleson KO , Kloner RA , *et al* Reperfusion injury induces apoptosis in rabbit cardiomyocytes. J Clin Invest. 1994; 94: 1621–8. 7929838 10.1172/JCI117504 PMC295322

[19] Scarabelli T , Stephanou A , Rayment N , *et al* Apoptosis of endothelial cells precedes myocyte cell apoptosis in ischemia/reperfusion injury. Circulation. 2001; 104: 253–6. 11457740 10.1161/01.cir.104.3.253

[20] Olivetti G , Abbi R , Quaini F , *et al* Apoptosis in the failing human heart. N Engl J Med. 1997; 336: 1131–41. 9099657 10.1056/NEJM199704173361603

[21] Ferrandi C , Ballerio R , Gaillard P , *et al* Inhibition of c‐Jun N‐terminal kinase decreases cardiomyocyte apoptosis and infarct size after myocardial ischemia and reperfusion in anaesthetized rats. Br J Pharmacol. 2004; 142: 953–60. 15210584 10.1038/sj.bjp.0705873 PMC1575119

